# Staphylococcus aureus surgical site infection following unilateral biportal endoscopic spine surgery: a two-case report

**DOI:** 10.3389/fsurg.2026.1788679

**Published:** 2026-04-13

**Authors:** Chunlin Hong, Lingfeng Chen, Huinuan Chen, Yahui Lin, Hong Lin, Zhirong Huang, Xuena Liu, Shiming Lin

**Affiliations:** 1 Department of Infection Prevention and Control, Fujian Province Zhangzhou Municipal TCM Hospital, Affiliated Hospital of Fujian University of Traditional Chinese Medicine, Zhangzhou, China; 2 Department of Surgery, Fujian Province Zhangzhou Municipal TCM Hospital, Affiliated Hospital of Fujian University of Traditional Chinese Medicine, Zhangzhou, China; 3 Department of Clinical Laboratory, Fujian Province Zhangzhou Municipal TCM Hospital, Affiliated Hospital of Fujian University of Traditional Chinese Medicine, Zhangzhou, China; 4 Hospital Infection Prevention and Control Committee, Fujian Province Zhangzhou Municipal TCM Hospital, Affiliated Hospital of Fujian University of Traditional Chinese Medicine, Zhangzhou, China

**Keywords:** case report, infection prevention, staphylococcus aureus, surgical site infection, unilateral biportal endoscopy

## Abstract

**Background:**

Surgical site infection (SSI) is a serious complication of spinal surgery, including minimally invasive unilateral biportal endoscopic (UBE) procedures. Staphylococcus aureus (S. aureus) is a leading cause of such infections. This report analyses two cases of S. aureus SSI following UBE surgery.

**Case presentation:**

Two patients (one male and one female) aged 56 underwent elective UBE surgery. Both patients had inadequate preoperative skin preparation. The cases were complicated by intraoperative fluid leakage, which led to soaked surgical drapes. The second case also involved prolonged operative time and failure of postoperative wound care, with the patient performing unsterile dressing changes at home. Both patients developed deep SSIs caused by S. aureus, as confirmed by culture. This required readmission, endoscopic debridement and targeted antibiotic therapy.

**Discussion:**

The differences in the antibiotic susceptibility profiles of the two cases suggest that the SSIs were likely caused by the patients’ own colonising flora. Key risk factors identified include inadequate skin preparation, intraoperative fluid leakage, prolonged surgery and breaches in postoperative care. These factors likely facilitated bacterial ingress and infection. In response, the institution implemented three key measures: direct nurse-led preoperative skin cleansing; increased surgical draping layers to prevent fluid saturation; and exclusive physician-performed postoperative dressings. Following these interventions, no new SSI cases were observed for over a year.

**Conclusion:**

SSI after UBE surgery is multifactorial. A comprehensive strategy that addresses preoperative, intraoperative and postoperative protocols is crucial for prevention. The simple, targeted interventions described here effectively mitigated the risk of infection in our subsequent practice.

## 1 Introduction

Surgical site infection (SSI) remains a particularly challenging complication during the early postoperative phase of spinal procedures. A comprehensive meta-analysis of 32 studies involving over 80,000 patients reported a pooled SSI incidence ranging from 0.7% to 12.0%. This varied significantly based on surgical technique and underlying patient comorbidities (1). A subsequent prospective cohort study analysing 1,145 spinal surgeries identified specific risk factors through multivariate logistic regression, including diabetes, obesity, and operation times exceeding three hours (2). These quantitative findings, derived from robust statistical analyses across different study designs and timeframes, emphasise the multifactorial nature of SSI pathogenesis and identify the populations at greatest risk.

Unilateral Biportal Endoscopic (UBE) surgery is a minimally invasive procedure for the spine that utilises two small portals: one for an endoscope and another for surgical instruments. This technique enables effective decompression for conditions such as lumbar disc herniation and spinal stenosis. As minimally invasive techniques such as UBE spine surgery become more widely adopted, there is an increasing need to understand the unique risk factors and microbiological profiles associated with these procedures. Although UBE surgery offers advantages such as reduced tissue trauma and faster recovery, it is not immune to infectious complications. To date, the true incidence of SSIs in UBE surgery remains understudied, with only a few researchers having documented cases of SSIs following UBE procedures (3, 4).

Staphylococcus aureus (S. aureus), particularly the methicillin-resistant strain (MRSA), is a leading cause of SSIs in orthopaedic surgeries, including spinal procedures (5). Unlike S. epidermidis, which is often a contaminant from skin flora, S. aureus is associated with more virulent, rapidly progressing infections. Common risk factors for SSIs include advanced age, obesity, diabetes, prolonged operative time and inadequate perioperative management (6, 7). However, the role of environmental and patient-related factors, such as poor wound care, surgical incision contamination, and compromised immune status, in facilitating S. aureus colonisation and subsequent infection, warrants further attention.

This study retrospectively analysed two cases to explore the potential aetiology of S. aureus SSI following UBE surgery. Both patients were admitted to a public tertiary hospital for surgery by an experienced orthopaedic surgeon.

## 2 Case presentation

### 2.1 Case 1

The first case involved a 56-year-old man with no history of hypertension, diabetes mellitus, infection or obesity, who underwent elective unilateral biportal endoscopic (UBE) L4/5 discectomy and spinal canal decompression. Notably, he did not receive preoperative antibiotic prophylaxis and his preoperative skin preparation was limited to verbal instructions. The surgery itself lasted two hours and 45 min. A critical intraoperative event was the observation of fluid leakage at the end of the procedure, resulting in the surgical drapes and the patient's clothing becoming soaked. He was discharged on the fourth postoperative day, but was readmitted just one day later with classic signs of a SSI, including redness, pain and purulent discharge from the wound. Upon readmission, cultures from the wound and blood confirmed a S. aureus infection. He required a second endoscopic procedure for irrigation and debridement, followed by treatment with vancomycin and then linezolid. Following discharge the second time, the surgical wound was clean, dry and intact, showing no signs of exudate or pus. The intraoperative fluid leak is considered a major contributing factor in the introduction of skin flora into the deep surgical site.

### 2.2 Case 2

The second case concerned a 56-year-old woman with a history of osteosarcoma following chemotherapy and rheumatoid arthritis, which suggests an immunocompromised state. She underwent a UBE procedure involving L4/5 discectomy and spinal canal decompression without receiving preoperative antibiotic prophylaxis, and her preoperative skin preparation could not be verified. Her surgery was notably prolonged, lasting over four hours. Additionally, this case involved intraoperative fluid leakage, resulting in the surgical drapes becoming saturated. Following discharge on the third postoperative day, she failed to adhere to medical instructions by not attending a local clinic for professional wound care, instead performing dressing changes herself at home. Within five days, she presented with redness, swelling and pain at the surgical incision site, accompanied by systemic symptoms such as fever and chills. Readmission and culture confirmed a S. aureus infection. She required endoscopic debridement and vancomycin therapy for treatment. The surgical incision healed well after the second discharge, with no exudate, purulent discharge or other complications present. This case highlights the convergence of several high-risk factors: significant comorbidities in the patient, prolonged surgery and critical failure in postoperative wound care, which likely introduced the pathogen.

Table 1 shows the epidemiological and clinical profiles of the two patients.

**Table 1:** Basic information of two patients.

Patient	Case 1	Case 2
Age	56 years	56 years
Sex	Male	Female
BMI	25.23 kg/m^2^	28.58 kg/m^2^
Osteosarcoma post-chemotherapy	No	Yes
Rheumatoid arthritis	No	Yes
Segment	L4/5	L4/5
Operative time	165 min	250 min
Blood loss	30 ml	50 ml
Skin preparation	Unreliable	Unreliable
Fluid leakage	Yes	Yes
Operating room	OR 11	OR 11
Operating date	2024-01-19	2024-02-02
Dates of first hospitalization	2024-01-17 to 2024-01-23	2024-02-01 to 2024-02-05
Dates of second hospitalization	2024-01-26 to 2024-02-09	2024-02-09 to 2024-03-15
Pathogenic bacteria	Staphylococcus aureus (sensitive to vancomycin)	Staphylococcus aureus (sensitive to vancomycin)

### 2.3 Epidemiological similarities and differences between the two cases

Both surgical procedures were performed by the same surgical team in the same operating theatre. The two patients were hospitalised in different rooms on the same ward, with their hospitalisation periods overlapping by six days. Their surgeries were scheduled 14 days apart (Case 1 on 19 January 2024 and Case 2 on 2 February 2024). During this period, the same team successfully performed four similar UBE procedures without any reported SSIs. At our institution, the post-use processing of reusable surgical instruments, including endoscopes, is conducted in strict accordance with International Organization for Standardization (ISO) Standard 15883-2:2023, following standardised procedures for collection, sorting, cleaning, disinfection, drying, inspection, maintenance, packaging, and sterilisation (using high- or low-temperature methods according to the specific requirements of the instruments). Sterilisation records for the surgical instruments used in both infected cases showed no irregularities, and subsequent bacterial cultures from the instrument surfaces did not detect S. aureus. Wound secretion cultures from both cases indicated S. aureus infections, though antibiotic susceptibility testing revealed differences in resistance profiles. The bacterial strain in Case 1 was resistant to penicillin G, tetracycline and trimethoprim-sulfamethoxazole. The strain in Case 2, meanwhile, was resistant to penicillin G, erythromycin and clindamycin. However, both strains remained sensitive to vancomycin. In accordance with the ‘Evidence-based clinical guideline for antibiotic prophylaxis in spine surgery (version 2013)’ and the ‘Evidence-based clinical guideline for the diagnosis and treatment of SSI in spinal trauma (version 2024)’ (8, 9), our facility treated both patients with SSI following UBE with a regimen of secondary surgical debridement combined with vancomycin anti-infective therapy, achieving favourable therapeutic outcomes. The pathogenic bacteria and antimicrobial susceptibility test results for the two patients are presented in Table 2. The treatment timelines for the two cases are presented in Table 3.

**Table 2:** Pathogenic bacteria and antimicrobial susceptibility test.

Antibiotic	Case1	Case2
Pathogenic bacteria	Staphylococcus aureus	Staphylococcus aureus
Penicillin G	MIC ≥ 0.5(R)	MIC ≥ 0.5(R)
Cefoxitin screen	Negative	Negative
Clindamycin induction (D-test)	Negative	Positive
Clindamycin	MIC ≤ 0.25(S)	MIC ≤ 0.25(R)
Nitrofurantoin	MIC = 32(S)	MIC = 32(S)
Tigecycline	MIC = 0.25(S)	MIC ≤ 0.12(S)
Tetracycline	MIC ≥ 16(R)	MIC ≤ 1(S)
Quinupristin/Dalfopristin	MIC = 0.5(S)	MIC ≤ 0.25(S)
Vancomycin	MIC ≤ 0.5(S)	MIC ≤ 0.5(S)
Linezolid	MIC = 2(S)	MIC = 2(S)
Erythromycin	MIC ≤ 0.25(S)	MIC ≥ 8(R)
Ciprofloxacin	MIC = 1(S)	MIC ≤ 0.5(S)
Levofloxacin	MIC = 1(S)	MIC ≤ 0.12(S)
Rifampin	MIC ≤ 0.5(S)	MIC ≤ 0.5(S)
Moxifloxacin	MIC ≤ 0.25(S)	MIC ≤ 0.25(S)
Trimethoprim/Sulfamethoxazole	MIC ≥ 320(R)	MIC ≤ 10(S)
Oxacillin	MIC = 0.5 μg/ml(S)	MIC ≤ 0.25 μg/ml(S)
Gentamicin	MIC ≤ 0.5 μg/ml(S)	MIC ≤ 0.5 μg/ml(S)

MIC, minimal inhibitory concentration (μg/ml); S, sensitive; R, resistant.

**Table 3:** The treatment timelines for the two cases.

Key time points	Case1	Case2
Initial surgery	2024-01-19	2024-02-02
Onset of infection symptoms	2024-01-24	2024-02-07
Bacterial culture sampling	2024-01-26	2024-02-09
Second surgery	2024-01-27	2024-02-17
Vancomycin aadministration	2024-01-28 to 2024-02-09	2024-02-11 to 2024-02-21
Dates of first hospitalization	2024-01-17 to 2024-01-23	2024-02-01 to 2024-02-05
Dates of second hospitalization	2024-01-26 to 2024-02-09	2024-02-09 to 2024-03-15

### 2.4 Diagnostic basis, diagnostic challenges, and differential diagnosis

In both cases, SSI was diagnosed through cultures of wound and blood samples, both of which identified S. aureus. The delayed presentation of symptoms after hospital discharge complicated the diagnosis. Additionally, extensive environmental sampling failed to identify a direct source of S. aureus, which made pinpointing the origin of the infection difficult. The primary diagnostic challenge was to distinguish SSI from normal postoperative inflammatory responses or other causes of systemic symptoms. The presence of purulent drainage in Case 1 and systemic symptoms, including fever and chills in Case 2, helped to differentiate infection from routine postoperative recovery.

### 2.5 Improvements and outcomes

Following analysis of the aforementioned two cases, infection control staff required the surgical team to make partial adjustments to the perioperative management details for subsequent UBE surgeries. Firstly, the importance of preoperative skin preparation was emphasised: nurses were required to cleanse the surgical site directly in the ward rather than relying on the patient to do so themselves. Secondly, the number of draping layers around the incision was increased to more than eight to prevent fluid saturation. Thirdly, a structured plan was established whereby in-house physicians performed or scheduled all postoperative wound dressings, including for discharged patients. Subsequent follow-up has confirmed an absence of new SSI cases in the 359-strong UBE surgery cohort since these improvements were introduced in March 2024. In our institution, the SSI incidence rate for all type I incision surgeries was 0.34% in 2024, compared to 0.23% in 2025. Direct comparison shows that, following implementation of the aforementioned measures, the current SSI incidence rate for UBE surgeries at our hospital is significantly lower than for other surgical procedures.

## 3 Discussion

SSI remains a significant complication following spinal procedures, including minimally invasive techniques such as unilateral biportal endoscopic (UBE) surgery. SSI pathogenesis typically involves bacterial invasion through compromised surgical barriers, followed by colonisation and biofilm formation on necrotic tissue or implants (5). Host defence mechanisms may be impaired by surgical stress, comorbidities and the local inflammatory response, creating a favourable environment for microbial proliferation (10).

The two cases presented herein illustrate how a combination of recognised risk factors and procedure-specific vulnerabilities can lead to serious postoperative infections. Both patients developed deep SSIs caused by S. aureus, a virulent pathogen that is well-known for its role in orthopaedic device-related infections. The organism's ability to express adhesins, such as microbial surface components recognising adhesive matrix molecules (MSCRAMMs), facilitates attachment to host tissues and implants, while its capacity to form biofilms significantly reduces its susceptibility to antibiotics (11). The divergent antibiotic resistance profiles strongly suggest that the two S. aureus infections originated from distinct bacterial strains rather than a common source, indicating different genetic lineages.

Multiple established risk factors were evident in these cases. Advanced age, prolonged operative time and inadequate preoperative skin preparation all significantly increased the risk of infection (1, 12). Case 2 further demonstrated that impaired immune function may compound this risk (12). Intraoperative fluid leakage, which compromised barrier protection and likely facilitated microbial ingress through capillary action, was particularly noteworthy in both cases (3). This mechanism is analogous to sweat contamination mechanisms.

It is well established that a significant proportion of the general population is asymptomatically colonised with S. aureus, which serves as a major reservoir for subsequent infection (13). Surgical procedures that breach the skin barrier can allow these colonising strains to invade the sterile surgical site. Although preoperative screening for S. aureus was not performed in our cases, this mechanism is highly consistent with the findings. In both Case 1 and Case 2, intraoperative fluid leakage likely facilitated the translocation of the patient's own colonising flora. In Case 2, the patient's immunocompromised state, coupled with a breach in sterile technique during home wound care, created an opportunity for her endogenous bacteria to cause infection. Therefore, the combination of patient-specific colonisation and procedural factors that compromise the surgical barrier best explains the onset of these two genetically distinct SSIs.

Our institutional response involved implementing evidence-based modifications that are aligned with the current understanding of SSI prevention. Direct nursing involvement in preoperative skin preparation is a critical first line of defence against bacterial colonisation. This practice is more effective than relying on patients to care for themselves and directly targets reducing the resident skin microbial burden, which is a primary source of pathogens in SSIs. Alcohol-based antiseptics are particularly effective against skin colonisers such as S. aureus. This protocol aligns with World Health Organization (WHO) guidelines emphasising the superiority of professionally applied antiseptic techniques over verbal instructions alone in significantly lowering SSI rates (14). Our institution reaffirms its stringent regulations and protocols regarding the selection and use of skin disinfectants. For surgical incision disinfection, tincture of iodine stock solution is applied directly to the skin. After allowing it to dry slightly, 70%–80% ethanol is used for deiodination. For clean incisions, the disinfection area should extend by at least 15 cm beyond the surgical field, with wiping performed from the centre to the periphery. Reinforced draping creates a more reliable fluid-resistant barrier, which is particularly important in UBE procedures involving continuous irrigation. The primary function of surgical drapes is to create a sterile barrier and manage fluid. Enhancing the draping system's fluid resistance directly mitigates the risk of capillary action, which can draw skin bacteria from non-sterile areas into the surgical site. Research has demonstrated that using reinforced, high-quality draping materials is significantly associated with reducing surgical wound infections (15). Exclusive physician-performed wound care ensures the correct technique is used and that infections are detected early, which is crucial for preventing disease progression. This measure guarantees that dressings are changed under aseptic conditions by trained staff, preventing the kind of exogenous contamination that occurred in the second case, where unsterile home care led to infection. Furthermore, it facilitates the early detection of complications. Previous studies have demonstrated that implementing a standardised wound care protocol can significantly reduce the incidence of SSIs, highlighting the critical importance of standardised nursing procedures (16). Following these improvements, no further UBE-related SSIs have occurred, indicating that the aforementioned perioperative measures are sufficient to resolve the issue. These measures are straightforward, yet they were not fully implemented in previous practice, largely due to a lack of awareness of infection risk among staff, which compromised implementation. These two UBE-related SSIs have served as a wake-up call, prompting us to heighten our vigilance against such nosocomial infections, and to re-examine the rationality and adequacy of our workflows and their implementation.

This case report provides a detailed clinical account of SSI after UBE. Comprehensive microbiological and environmental investigations helped to exclude sources of exogenous contamination. The longitudinal follow-up after implementing targeted interventions adds practical value. However, the study is limited by its small sample size (*n* = 2), which precludes statistical generalisation, and the absence of a control group, which limits causal inference regarding intervention effectiveness. Compared with previous reviews (3, 4), our findings are consistent with the identified risk factors. Notably, our extensive environmental sampling failed to identify S. aureus, which supports the hypothesis that SSIs originated from the endogenous flora of the patients rather than from exogenous sources. This emphasises the critical importance of preoperative skin antisepsis in UBE procedures.

Although Staphylococcus epidermidis often dominates discussions of implant-related infections due to its biofilm-forming capabilities, our cases demonstrate that S. aureus poses an equal, if not greater, risk in UBE procedures (17). The successful resolution of both cases through endoscopic debridement and antibiotic therapy emphasises the importance of combining mechanical biofilm disruption with appropriate antimicrobial coverage (18). Future preventive strategies could benefit from novel approaches, such as antibiotic-coated implants and enhanced surgical site protection systems designed specifically for endoscopic procedures (19). Additionally, some researchers have attempted to sample and culture the nasal cavities, throats and perineums of preoperative patients to analyse S. aureus colonisation status at these sites (20). We are considering implementing this approach in our clinical practice by preserving microbiological records of these areas before surgery. This would facilitate traceability and help identify the source of a SSI, if one were to occur.

## 4 Conclusion

These cases demonstrate that SSIs following UBE are typically multifactorial in origin, rather than resulting from isolated breaches. An effective prevention strategy must address patient-related, procedure-specific and postoperative care factors. The implemented measures are simple yet effective modifications to perioperative protocols that have been shown to provide significant clinical benefits in our practice.

## Data Availability

The original contributions presented in the study are included in the article/Supplementary Material, further inquiries can be directed to the corresponding authors.
